# Elucidating the Mechanism of Ambient-Temperature Aldol Condensation of Acetaldehyde on Ceria

**DOI:** 10.1021/acscatal.1c01216

**Published:** 2021-06-30

**Authors:** Suman Bhasker-Ranganath, Md. Saeedur Rahman, Chuanlin Zhao, Florencia Calaza, Zili Wu, Ye Xu

**Affiliations:** † Cain Department of Chemical Engineering, Louisiana State University, Baton Rouge, Louisiana 70803, United States; ‡ Instituto de Desarrollo Tecnoloǵico para la Industria Química (INTEC), CONICET-UNL, Santa Fe 3000, Argentina; § Chemical Sciences Division, Oak Ridge National Laboratory, Oak Ridge, Tennessee 37831, United States

**Keywords:** aldol condensation, CeO_2_, acetaldehyde, crotonaldehyde, DRIFTS, density functional theory, reaction mechanism

## Abstract

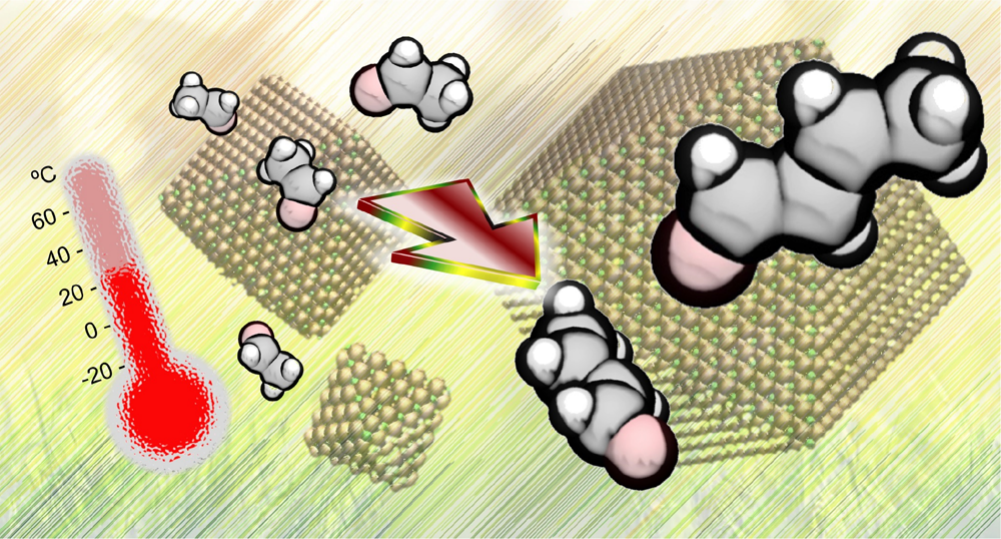

Using in situ diffuse reflectance infrared Fourier transform spectroscopy (DRIFTS) and density functional theory (DFT) calculations, we conclusively demonstrate that acetaldehyde (AcH) undergoes aldol condensation when flown over ceria octahedral nanoparticles, and the reaction is desorption-limited at ambient temperature. *trans*-Crotonaldehyde (CrH) is the predominant product whose coverage builds up on the catalyst with time on stream. The proposed mechanism on CeO_2_(111) proceeds via AcH enolization (i.e., α C–H bond scission), C–C coupling, and further enolization and dehydroxylation of the aldol adduct, 3-hydroxybutanal, to yield *trans*-CrH. The mechanism with its DFT-calculated parameters is consistent with reactivity at ambient temperature and with the kinetic behavior of the aldol condensation of AcH reported on other oxides. The slightly less stable *cis*-CrH can be produced by the same mechanism depending on how the enolate and AcH are positioned with respect to each other in C–C coupling. All vibrational modes in DRIFTS are identified with AcH or *trans*-CrH, except for a feature at 1620 cm^–1^ that is more intense relative to the other bands on the partially reduced ceria sample than on the oxidized sample. It is identified to be the C=C stretch mode of CH_3_CHOHCHCHO adsorbed on an oxygen vacancy. It constitutes a deep energy minimum, rendering oxygen vacancies an inactive site for CrH formation under given conditions.

## Introduction

Energy and chemical production from renewable sources including biomass has been a major driver for both academic and industrial research for the past two decades.^1−4^ Lignocelluloses can be deconstructed via physiochemical or biological methods, such as pyrolysis or hydrolysis, to create mixtures composed of small organic oxygenates that then need to be upgraded to fuel or higher value products. Reactions that can form C–C bonds and create larger organic compounds from smaller ones are desirable.^5−9^ Aldol condensation, a type of reaction commonly used in organic synthesis to achieve C–C bond formation, has been identified also as a useful route in catalytic biomass upgrading for this purpose. Having a catalyst that can actively and selectively catalyze this reaction will be key to an alternative, biomass-based chemical industry.

There is a consensus that the mechanism of aldol condensation generally involves the formation of an enol or enolate through dehydrogenation of the α carbon in a carbonyl compound forming a nucleophilic C center, which then attacks the electrophilic C in the C=O of another carbonyl compound, followed by the elimination of a H_2_O molecule. This can occur between two molecules of the same kind (self-condensation) or different kinds (cross condensation). Obviously, the reaction involves more details than is outlined above. To understand the aldol condensation of aldehydes and ketones, as well as related reactions such as the Guerbet reaction, many studies have been carried out on a variety of solid compounds, including CeO_2_, ZrO_2_, TiO_2_, Al_2_O_3_, MgO, mixed oxides, layered double hydroxides, and amorphous aluminophosphates.^10−20^ Much effort has been made to improve catalytic performance, through modifying the availability or strength of acid and base sites, geometry and coordination of metal ions, or extent of reduction or through mixing different oxides.^12,18,21−25^ The use of different oxides and different reaction conditions, and possibly co-catalysts (e.g., Cu, Pd)^15,26^ or co-reactants (e.g., H_2_, alcohols),^17,26,27^ complicates a comprehensive understanding of the mechanism of this catalytic reaction and the factors that control its activity and selectivity. Different steps including enolization (C–H bond scission at the α position to the carbonyl group) and C–C coupling have been found to be rate-determining in the literature, with different reaction orders and either the presence or absence of the kinetic isotope effect reported.

The aldol condensation of acetaldehyde (CH_3_CHO, abbreviated hereafter as AcH) is one of the simplest aldol condensation reactions because AcH is the smallest carbonyl compound that can enolize. Reactivity of AcH has been studied on polycrystalline ceria,^11,14^ shape-defined ceria nanoparticles (CeNPs),^28−30^ and oriented ceria thin film surfaces.^31−33^ An apparent pressure gap is evidenced by reports in the literature. Under a finite AcH partial pressure, a variety of C_2–4_ (and sometimes aromatic) species are produced. The highest product selectivity always goes to crotonaldehyde (i.e., 2-butenal, CH_3_CHCHCHO; abbreviated hereafter as CrH), suggesting it to be the primary product. The selectivity to CrH ranging from ca. 20 to 100% has been reported on ceria.^11,28,30,34^

In ultrahigh vacuum (UHV) conditions, on the other hand, CrH is rarely observed. The adsorption of AcH on thin crystalline films of CeO_2_(111)^31,32^ and CeO_2_(100)^33^ has been studied using temperature-programmed desorption (TPD) in UHV. CrH was observed as a minor product on oxidized CeO_2_(100) but not at all on partially reduced CeO_2_(100). On CeO_2_(111), while the formation of the enolate of AcH was conclusively established,^32^ no formation of CrH was observed regardless of extent of reduction. More recently, a double-ramp procedure was proposed that did produce CrH in the TPD of AcH on partially reduced CeO_2_(111).^35^ We proposed oxygen vacancy dimers to be the active site for C–C coupling in UHV, the formation of which was made possible through the double ramp procedure.

In the present work, we further the investigation of the aldol condensation of AcH on shape-controlled ceria nano-octahedra (referred to hereafter as o-CeNPs). o-CeNPs have been shown to be more selective for CrH formation than other shapes such as nanocubes and nanowires.^28^ They exhibit primarily (111) facets, which facilitates comparison with previous works based on CeO_2_(111) thin film surfaces.^31,32,35^ Infrared spectroscopy, in its many variations, has proven to be a powerful tool for elucidating the nature of reaction intermediates and mechanisms in heterogeneous catalysis.^36^ We use in situ diffuse reflectance infrared Fourier transform spectroscopy (DRIFTS) and density functional theory (DFT) calculations to demonstrate conclusive evidence for the ambient-temperature formation of CrH on CeO_2_(111). Unlike the previous study by Mann et al. that was based on oxidized o-CeNPs,^28^ here, we test both oxidized and partially reduced o-CeNPs. At extended times, the DRIFTS spectra for both samples look broadly similar, but subtle differences exist in how the various vibrational modes evolve with time. We propose reaction mechanisms based on DFT-calculated parameters, for both stoichiometric and oxygen vacancy sites on CeO_2_(111). The results are found to be consistent with the DRIFTS spectra and observed reactivity and offer a unified basis for rationalizing a range of different kinetic behaviors observed for the aldol condensation of AcH on various oxides. With this study, we aim to advance the fundamental understanding of aldol condensation reactions on oxide catalysts and contribute to the design of earth-abundant catalytic materials for this important type of reaction.

## Methods

### Experimental

o-CeNPs were prepared according to a previous procedure described in ref (37), which produced nano-octahedra around 100 nm in size. The in situ Fourier transform infrared (FTIR) study was conducted in a diffuse reflectance cell (Pike Technologies, Model HC-900, cell volume of ∼6 cm^3^) with a Nicolet Nexus 670 FTIR spectrometer using an MCT/A detector with a spectral resolution of 4 cm^–1^. For the oxidized sample, o-CeNPs were pretreated by heating to 673 K (10 K/min) and holding for 1 h in a gas stream of 5% O_2_/He (25 mL/min). The sample was then cooled down in 5% O_2_/He flow to 373 K and switched to He to further cool to ambient temperature, before background spectrum collection. A flow of 0.5% AcH/He (25 mL/min) was introduced to the sample, and continuous spectra (64 scans each) were collected over 30 min. The reactant flow and the reactor were maintained at ambient temperature during the adsorption experiments. In another experiment, a partially reduced sample was made by treating o-CeNPs at 673 K in 4% H_2_/He for 1 h following the oxidation treatment described above. The sample was then cooled down in 4% H_2_/He flow to 373 K and purged with He while cooling down to ambient temperature. The adsorption of AcH and characterization with DRIFTS were conducted in the same procedure as applied to the oxidized sample. All DRIFTS spectra shown below are difference spectra by subtracting out the background spectrum collected at ambient temperature for each sample before the adsorption of AcH.

### Theoretical

Spin-polarized periodic DFT calculations were performed using the Vienna Ab initio Simulation Package (VASP)^38^ in the generalized gradient approximation (GGA-PW91).^39^ The potentials due to the nuclei and core electrons (Ce(4f5s5p5d6s), O(2s2p), C(2s2p), H(1s)) were described by the projector augmented wave (PAW) method.^40,41^ The valence electrons were expanded in a plane wave basis set with a 400 eV kinetic energy cutoff. In addition to GGA-PW91, the optB86b-vdW functional^42^ was used to evaluate the van der Waals (vdW) contributions to the reaction energetics. OptB86b-vdW is known to overpredict adsorption energies for chemisorbed species on metals,^43^ but there have not been adequate experimental data to verify its accuracy for predicting adsorption energies on oxides.

The surface model for CeO_2_(111) was a slab consisting of three O–Ce–O tri-layers (i.e., a total of nine atomic layers) with a (2 × 2) surface unit cell, which corresponded to 1/4 monolayer (ML) of coverage for each adsorbate per unit cell. The periodic slab was separated by ca. 12 Å of empty space in the *z* direction. The adsorbates were placed on one side of the slab only, with dipole corrections applied in the *z* direction.^44^ A Γ-centered 2 × 2 × 1 Monkhorst-Pack k-point grid was used to sample the surface Brillouin zone.^45^ The topmost tri-layer and all adsorbates thereon were relaxed, while the bottom two tri-layers were fixed in their bulk positions. Molecules in the gas phase were optimized in a 15 × 15 × 15 Å^3^ simulation cell with dipole corrections applied in all three directions.

The adsorption energy of an adsorbate was calculated as Δ*E*_ads_ = *E*_total_ – *E*_slab_ – *E*_gas_, where *E*_total_ is the energy of the slab with adsorbates, *E*_slab_ is the energy of the clean slab without any adsorbate, and *E*_gas_ is the energy of the adsorbate molecule in a neutral state in the gas phase. Thus, less negative Δ*E*_ads_ corresponds to weaker adsorption. The climbing image nudged elastic band (CI-NEB)^46,47^ and dimer methods^48^ were used to determine the minimum energy pathway of each elementary step and the associated transition state. The activation barrier of an elementary step was calculated as *E_a_* = *E*_TS_ – *E*_IS_, where *E*_TS_ is the energy of transition state (TS) and *E*_IS_ is the energy of initial state (IS). Optimization of adsorption as well as TS geometries was converged to below 0.03 eV/Å in all degrees of freedom for all relaxed atoms.

Vibrational modes and frequencies were calculated in the harmonic approximation using a finite difference approximation of the dynamical matrix with a displacement of ±0.01 Å. No scaling factor was applied to the calculated frequencies. Each TS was verified to possess only one vibrational mode with a negative curvature in the direction of the bond breaking or forming process. Infrared (IR) spectra of adsorbates were simulated using the Atomic Simulation Environment (ASE).^49^ The IR intensities of the vibrational modes were calculated from a finite difference approximation of the gradient of the dipole moment in the *z* direction with a displacement of ±0.01 Å. Calculated normal modes were assigned to specific vibrations by visual inspection. The free energy correction to the energy of an adsorbed species (δ*G*) was calculated as described in ref (50).

The DFT + *U* method introduced by Dudarev et al.^51^ was used to offset the tendency of GGA to delocalize strongly correlated electrons. It penalizes non-integral occupation of certain orbitals (in this case the 4*f* orbitals of Ce) through an effective on-site interaction parameter. While a *U*_eff_ of 4–5 eV has been widely used to achieve a proper description of the electronic structure of reduced Ce centers, our studies of AcH reactions on CeO_2_(111) found that smaller *U*_eff_ values gave more accurate predictions of surface reaction kinetics, as gauged by peak desorption temperatures in TPD.^32,35,52^ Small *U*_eff_ values (≲ 2 eV) were also recommended by other authors based on comparison with the experimental reaction and adsorption energies involving ceria,^53−55^ albeit at the cost of not being able to sufficiently localize the excess electrons associated with oxygen vacancies.^56^ Below, we continue to report energies obtained with *U*_eff_ = 2 eV. The lattice constant of bulk CeO_2_ was calculated to be 5.476 and 5.452 Å at *U*_eff_ = 2 eV according to GGA-PW91 and optB86b-vdW, respectively, which compared closely with the experimental value of 5.41 Å.^57,58^

## Results

### Flow Reactor and DRIFTS Studies

**Figure 1:**
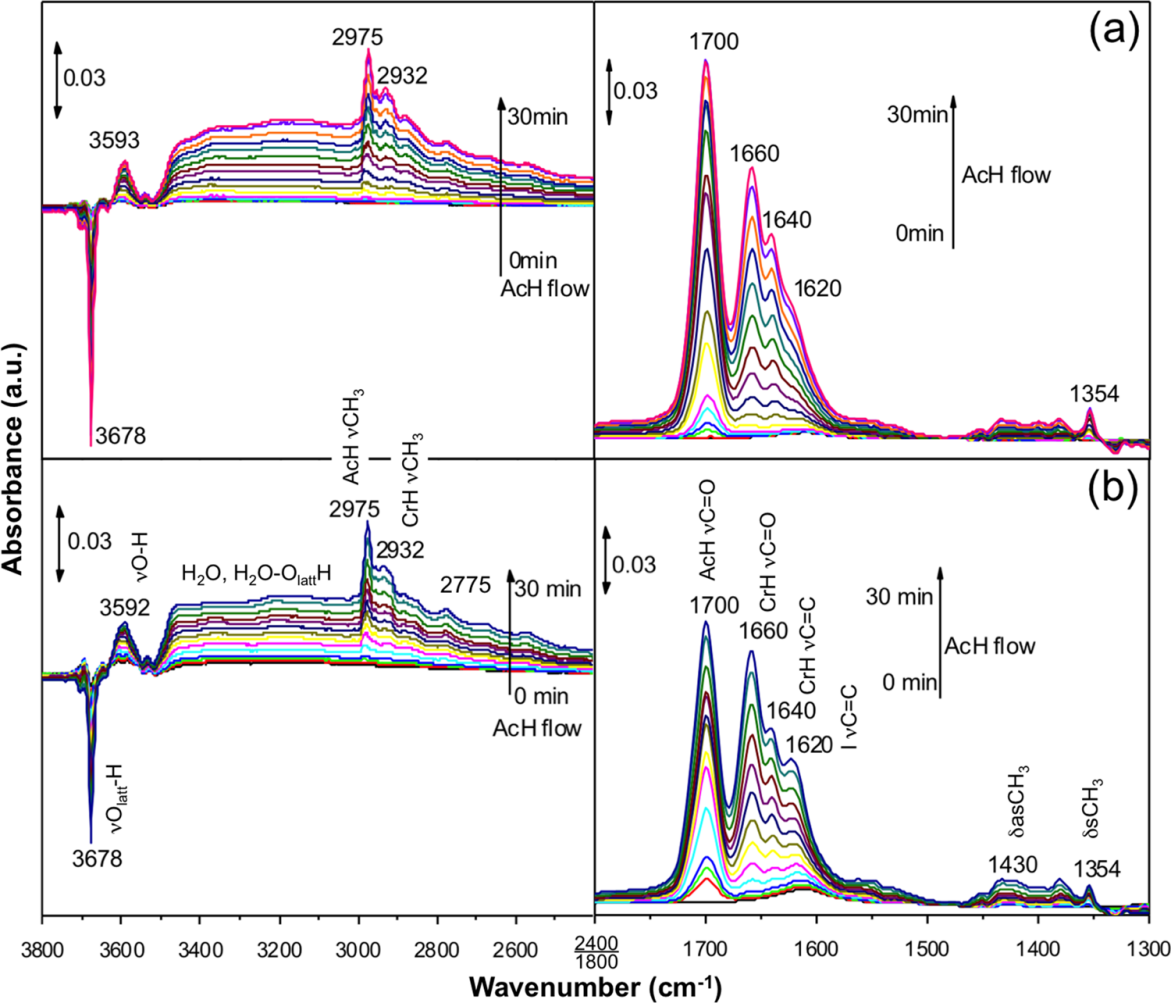
In situ DRIFTS spectra of 0.5% AcH/He flown at 25 mL/min over (a) oxidized o-CeNPs and (b) partially reduced o-CeNPs taken at 2 min intervals over a period of 30 min at room temperature. Peak assignments apply to both spectra; “I” refers to CH_3_CHOHCHCHO/V_O_; see text for detail.

The oxidized and partially reduced o-CeNPs were tested separately in the same flow reactor under identical reaction conditions as described in the Methods section. DRIFTS spectra were taken at 2 min intervals over a period of 30 min at room temperature. The resulting spectra in the 1300–1800 cm^–1^ region (Figure 1, right panels), which is expected to include both νC=O and νC=C stretching modes, and in the 2400–3800 cm^–1^ region (Figure 1left panels), which is expected to include water, OH, and CH*_x_* stretching modes, are broadly similar for the two samples at extended times. The same set of peaks is observed, with slight differences in their relative intensities. The most intense peak in 1300–1800 cm^–1^ is located at 1700 cm^–1^, which we assign to the νC=O mode in AcH.^32^ The other two major peaks at 1660 and 1640 cm^–1^ (with a shoulder at 1620 cm^–1^) and two minor ones at 1430 and 1354 cm^–1^ have not appeared in a previous UHV experiment of AcH on CeO_2_(111).^32^

Raskó and Kiss performed AcH adsorption from the gas phase on polycrystalline CeO_2_ and detected CrH among the volatile products but did not conclusively assign any peaks in 1300–1800 cm^–1^ to CrH in FTIR.^14^ Young et al. assigned 1684 cm^–1^ on TiO_2_, 1674 cm^–1^ on hydroxyapatite, and 1719 cm^–1^ on MgO to the νC=O mode of CrH upon AcH adsorption but did not clearly identify any peak with the νC=C mode of CrH.^17^ Rekoske and Barteau dosed CrH on TiO_2_ and assigned to νC=O and νC=C of CrH the following frequencies: 1686 and 1636 cm^–1^ on anatase TiO_2_, and 1656 and 1602 cm^–1^ on rutile TiO_2_.^34^ Singh et al. dosed AcH on polycrystalline TiO_2_ and assigned the following peaks to CrH in FTIR: 1656 (νC=O), 1642 and 1630 (νC=C), 1442 (δ_as_CH_3_), and 1376 cm^–1^ (δ_s_CH_3_).^59^ Mann et al. assigned peaks in DRIFTS at 1660 and 1640 cm^–1^ to νC=O and νC=C of CrH, respectively, in the TPD of AcH on o-CeNPs,^28^ and we make the same assignments. The cluster of modes in 1350–1460 cm^–1^ could be due to symmetric and asymmetric bending of the methyl group in AcH, CrH, or reaction intermediates. Overall, the previous FTIR studies of AcH adsorption on ceria had limited resolution in this crucial frequency range.^14,28^ The peaks observed in our experiment are clearly resolved and parallel those reported by Singh et al.^59^ on TiO_2_. They did not explain why there should be two different νC=C modes for CrH.

The broad feature at 3000–3500 cm^–1^ is assigned to domains of aggregated water molecules, which build up with time on stream just like the CrH modes. The negative peak at 3678 cm^–1^ is due to the consumption of O_latt_–H species on ceria,^60^ which are prevalent on oxides. Interaction with the product water and oxygenate molecules via hydrogen bonding causes it to be red-shifted and subsumed into the broad feature at 3000–3500 cm^–1^, so its intensity decreases with time. The 3593 cm^–1^ band is likely also a νO–H stretch, which would suggest the formation of a new OH group upon the adsorption and reaction of AcH. The band at 2975 cm^–1^ is assigned to the methyl stretch in AcH, whereas the weaker one at 2932 cm^–1^ is assigned to the methyl stretch in CrH.^32,59^ Additional weaker features are visible in 2700–3000 cm^–1^.

While the two sets of spectra look similar, upon close inspection, subtle but clear differences can be identified in how the vibrational modes develop with time. In the first 2 min, even before the νC=O mode of AcH (1700 cm^–1^) begins to grow, a mode at 1610 cm^–1^ appears for both samples. On the oxidized o-CeNPs, the AcH mode overtakes this mode in the next 2–4 min, accompanied by the rise of the CrH modes (1660 and 1640 cm^–1^). On the partially reduced o-CeNPs, the 1610 cm^–1^ mode remains comparable in intensity to the 1700 cm^–1^ mode in the next few minutes. As time goes on, the 1610 cm^–1^ mode gradually blue-shifts to 1620 cm^–1^. Relative to the AcH and CrH modes, it remains somewhat stronger on the partially reduced o-CeNPs than on the oxidized o-CeNPs. This pattern suggests that the 1610–1620 cm^–1^ feature is due to a species that is associated with reduced sites and is formed just as rapidly as, or even more so than, CrH. Below, we use first-principles-based modeling to resolve the identities of the main features that are seen in DRIFTS.

### Proposed Mechanism on Stoichiometric CeO_2_(111)

**Figure 2:**
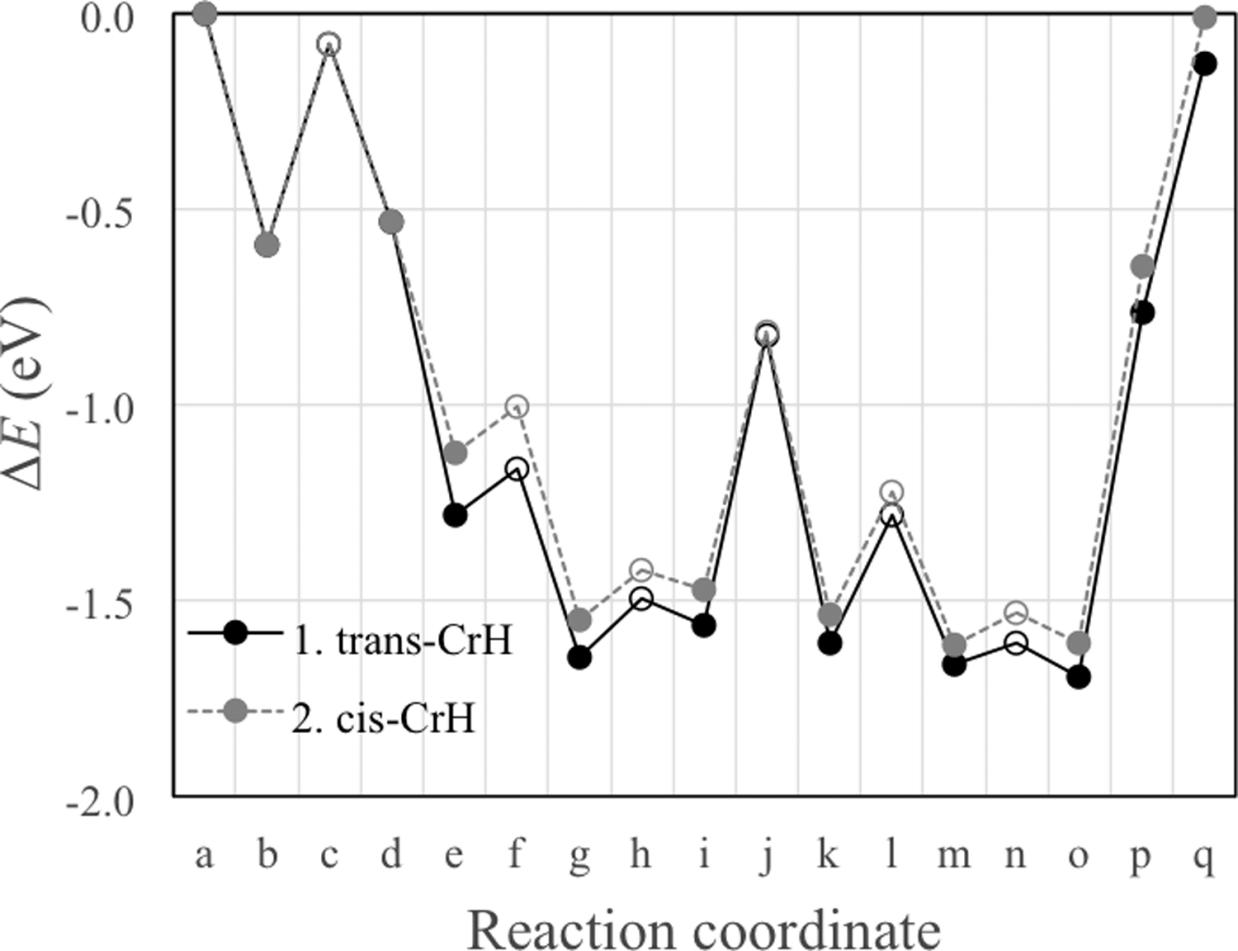
Reaction energy profiles for the proposed mechanism for the aldol condensation of AcH to form *trans*-CrH (solid line) and *cis-*CrH (dashed line) on stoichiometric CeO_2_(111). The states along the reaction coordinate are (a) AcH_(g)_, (b) the first adsorbed AcH; (c) TS of the first α C–H scission; (d) Enl + H; (e) the second AcH co-adsorbed with Enl + H; (f) TS of the C–C coupling of AcH and Enl; (g) CH_3_CHOCH_2_CHO + H; (h) TS of hydrogenation; (i) 3HBtL; (j) TS of the second α C–H scission; (k) CH_3_CHOHCHCHO + H; (l) TS of dehydroxylation; (m) CrH + OH + H; (n) TS of H_2_O formation; (o) CrH + H_2_O; (p) H_2_O + CrH_(g)_; and (q) CrH_(g)_ + H_2_O_(g)_. Filled dots represent stable intermediates, and open dots represent TSs.

**Figure 3:**
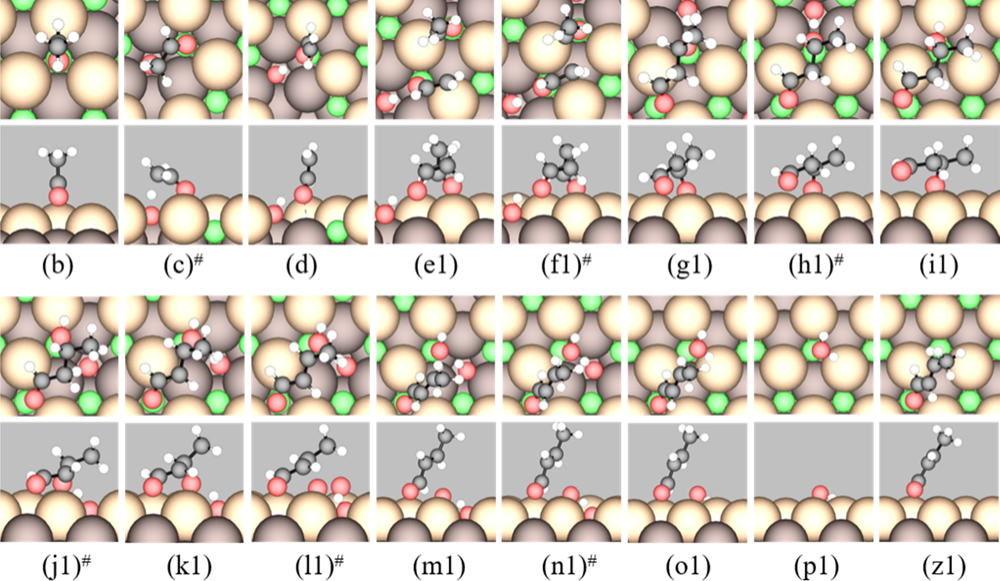
Top (upper panels) and side (lower panels) views of stable intermediates and TSs (labeled #) in the proposed mechanism for aldol condensation of AcH to *trans*-CrH on stoichiometric CeO_2_(111). The labels correspond to those in Table 1, except (z1). The states shown are (b) AcH; (c) TS of the first α C–H scission; (d) Enl + H; (e1) AcH + Enl + H; (f1) TS of the C–C coupling of AcH and Enl; (g1) CH_3_CHOCH_2_CHO + H; (h1) TS of hydrogenation; (i1) (R)-3HBtL; (j1) TS of the second α C–H scission; (k1) CH_3_CHOHCHCHO + H; (l1) TS of dehydroxylation; (m1) CrH + OH + H; (n1) TS of H_2_O formation; (o1) CrH + H_2_O; (p1) H_2_O; and (z1) *trans*-CrH. Color code: green = lattice Ce, light brown = surface lattice O, dark brown = subsurface lattice O, red = O in molecules, black = C, and white = H. Surface lattice O atoms bonded to C or H atoms in the molecules are considered part of the molecules.

The overall reaction in gas phase,
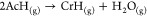
1

has a calculated reaction energy of −0.13 eV for *trans*-CrH (i.e., (E)-CrH, the more stable diastereomer of CrH) and −0.01 eV for *cis*-CrH (i.e., (Z)-CrH) according to both GGA-PW91 and optB86b-vdW. Based on previous insights,^35,59^ we propose a reaction mechanism consisting of the elementary steps listed in Table 1 for the aldol condensation of AcH to *trans*-CrH on stoichiometric (i.e., oxidized) CeO_2_(111). The DFT-calculated reaction energy profile is plotted in Figure 2, with snapshots of the reaction intermediates shown in Figure 3.

**Table 1:** DFT-Calculated Activation Barrier (*E*_a_, in eV) and Reaction Energy (Δ*E*_rxn_, in eV) for the Elementary Steps in the Proposed Mechanism for Aldol Condensation of AcH on Stoichiometric CeO_2_(111)^a^

label	elementary step	*E*_a_	Δ*E*_rxn_
*trans* Mechanism			
a → b	AcH_(g)_ → AcH	0^b^	–0.59^c^
b → c → d	AcH → Enl + H	0.52	+0.06
e1 → f1 → g1	AcH + Enl → CH_3_CHOCH_2_CHO (+ H)	0.12	–0.36
g1 → h1 → i1	CH_3_CHOCH_2_CHO + H → 3HBtL	0.15	+0.08
i1 → j1 → k1	3HBtL → CH_3_CHOHCHCHO + H	0.74	–0.05
k1 → l1 → m1	CH_3_CHOHCHCHO → CrH + OH (+ H)	0.33	–0.05
m1 → n1 → o1	OH + H → H_2_O (+ CrH)	0.10^d^	–0.03
o1 → p1	CrH → CrH_(g)_ (+ H_2_O)	0.93^b^	+0.93^c^
p1 → q	H_2_O → H_2_O_(g)_	0.63^b^	+0.63^c^
*cis* Mechanism			
e2 → f2 → g2	AcH + Enl → CH_3_CHOCH_2_CHO (+ H)	0.12	–0.42
g2 → h2 → i2	CH_3_CHOCH_2_CHO + H → 3HBtL	0.13	+0.08
i2 → j2 → k2	3HBtL → CH_3_CHOHCHCHO + H	0.66	–0.06
k2 → l2 → m2	CH_3_CHOHCHCHO → CrH + OH (+ H)	0.31	–0.08
m2 → n2 → o2	OH + H → H_2_O (+ CrH)	0.10^d^	+0.01
o2 → p2	CrH → CrH_(g)_ (+ H_2_O)	0.96^b^	+0.96^c^

a *E*_a_ and Δ*E*_rxn_ are based on GGA-PW91 electronic energies (*U*_eff_ = 2 eV) without ZPE correction. Step labels refer to Figure 2. Species shown in parentheses are co-adsorbed and are not directly involved in the given steps. Free sites are omitted from the description. Steps in the *cis* mechanism identical to those in the *trans* mechanism are omitted for brevity.

b Adsorption assumed to be barrier-less and desorption assumed to have no kinetic barrier in addition to the thermodynamic barrier.

c Corrected for vdW interactions based on optB86b-vdW results.

d Minimal calculated barrier, replaced with a value representing the OH diffusion barrier.

The mechanism is predicated on the acid–base bifunctional property of the ceria surface, where the acidic Ce cation stabilizes the carbonyl species through interaction with the carbonyl O, and the basic lattice O anion serves as the hydrogen abstraction site. It requires the availability of molecularly adsorbed AcH. In UHV, molecularly adsorbed AcH desorbs from CeO_2_(111) around 210 K^31^ and would not be available for further reaction at ambient temperature. Here, a flow of AcH maintains a finite AcH partial pressure (*p*_AcH_) that makes molecular AcH available for further reaction on the surface.

As previously reported,^32^ AcH adsorbs molecularly in an η^1^ configuration (Figure 3b), with the carbonyl O positioned on a threefold cation (3fc) site, which is located above a topmost Ce atom, and with the acyl H atom approaching a lattice O atom (O_latt_) at the surface. The Δ*E*_ads_ (which is taken to be the same as the Δ*E*_rxn_ for the AcH adsorption step a → b) is −0.30 (GGA-PW91) or −0.59 (optB86b-vdW) eV at 1/4 ML coverage. Likewise, an individual *tran*s-CrH molecule adsorbs stably in an η^1^ configuration on a 3fc site (Figure 3z1) with a Δ*E*_ads_ of −0.24 (GGA-PW91) and −0.60 (optB86b-vdW) eV, respectively. Thus, we apply corrections of +0.29 and +0.36 eV to the electronic energies of AcH and CrH in the gas phase, respectively, and then correct that of H_2_O in the gas phase accordingly to maintain the overall reaction energy (eq 1). The Δ*E*_ads_ are tabulated in Table S1 in the Supporting Information (SI). The resulting Δ*E*_ads_ for H_2_O agrees closely with the optB86b-vdW results reported in ref (61). The reaction energy profiles as calculated using GGA-PW91; using GGA-PW91 with the adsorption of AcH, CrH, and H_2_O corrected for vdW interactions; and using optB86b-vdW directly are compared in Figure S1 in the SI. It can be seen that GGA-PW91 and optB86b-vdW agree closely on the energetics of the surface-bound steps because the vdW contributions largely cancel out for the initial, transition, and final states for such steps. A difference of ca. 0.2 eV is introduced by state e, which we attribute to attractive interaction between AcH and its enolate (Enl) that is not captured by GGA-PW91.

**Scheme 1:**
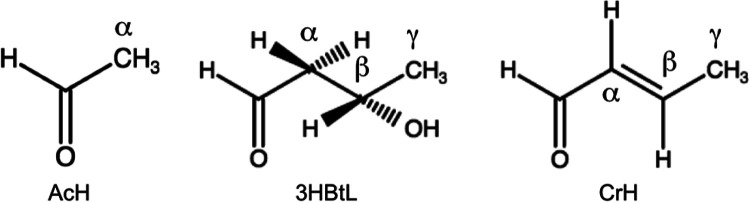
Assignments of C Atoms in Acetaldehyde (AcH), 3-Hydroxybutanal (3HBtL), and Crotonaldehyde (CrH)

Proceeding from the η^1^ state, one of the α C–H bonds in AcH (see Scheme 1 for the labeling of the C atoms in AcH, 3-hydroxybutanal (abbreviated as 3HBtL), and CrH) closest to the surface lengthens from 1.10 to 1.34 Å in the TS of enolization (Figure 3c), at a modest activation barrier of *E*_a_ = 0.52 eV (step b → c → d). The H atom is abstracted by the nearest O_latt_. The ease of AcH enolization can be attributed to the stabilization of the carbonyl O by coordination to lattice Ce, which allows the C=O double bond to re-hybridize to a single bond and the C–C single bond to re-hybridize to C=C to compensate for the loss of H. The activation barrier for enolization is nearly identical to the barrier for AcH desorption, which means that a finite *p*_AcH_ is needed not only to keep AcH on the surface but also to allow it to react in aldol reactions. To the extent that O_latt_ sites are needed to initiate the reaction by activating AcH, the more hydroxylated the surface is (e.g., as a result of synthesis), the less catalytically active it is expected to be overall.

A second AcH then co-adsorbs in the η^1^ state with the Enl (Figure 3e1), which is 0.16 eV more stable than if they are at infinite separation. C–C coupling occurs between the nucleophilic α C of the Enl and the electrophilic carbonyl C of the AcH, forming the intermediate adduct CH_3_CHOCH_2_CHO (Figure 3g1), with a small activation barrier of *E*_a_ = 0.12 eV. The distance of the C–C bond in the TS (Figure 3f1) is 2.23 Å, which reduces to 1.55 Å in the adduct that is typical of a C–C single bond.^32^ After the C–C coupling step, the interior O atom is stabilized through coordination to lattice Ce and a nearby H (dO–H = 1.66 Å).

The transformation that needs to be accomplished next includes the removal of another H atom on the α C and the removal of the interior O on the β C, which most likely would only be feasible after it is hydrogenated to OH. Several possible pathways exist, and a priori, it is not clear which one should be preferred. These are as follows: (i) direct (non-catalytic) intra-molecular H shift from α C to the interior O in CH_3_CHOCH_2_CHO; (ii) α H abstraction by O_latt_ in CH_3_CHOCH_2_CHO; and (iii) α H abstraction by O_latt_ following hydrogenation of the interior O by a surface-bound H to CH_3_CHOHCH_2_CHO (3HBtL). The energies of the respective C–H scission TSs are, relative to the last one, 0.69, 0.21, and 0.00 eV, respectively. Thus, we propose the mechanism to proceed through the formation of 3HBtL.

Transferring a surface-bound H atom to the interior O (step g1 → h1 → i1) has a small activation barrier (*E_a_* = 0.15 eV; Figure 3h1) for several reasons: (i) the H is a proton for all practical purposes while the O is negatively charged; (ii) the β C is over-coordinated; and (iii) the product, 3HBtL, is a closed-shell species (Figure 3i1). The following step (step i1 → j1 → k1), which involves the second C–H bond scission at the α position and transfer of the H atom to a nearby O_latt_ atom, has the highest activation barrier among the surface steps (*E_a_* = 0.74 eV). dC–H and dO–H are 1.40 and 1.29 Å, respectively, in the TS (Figure 3j1), suggesting that the adsorption configuration of 3HBtL is flexible enough to allow the α C to be favorably positioned with respect to an O_latt_ site, which facilitates the H abstraction step. Wang et al. showed that larger carbonyl compounds such as ketones and branched aldehydes tend to have larger enolization barriers than linear aldehydes on TiO_2_,^62^ which is consistent with our finding that C–H bond scission has a higher barrier in 3HBtL than in AcH on CeO_2_(111). Very recently, Li et al. also reported a higher *E_a_* of enolization for 3HBtL than for AcH on TiO_2_ calculated using DFT.^63^

All the pathways outlined above lead to the formation of CH_3_CHOHCHCHO (Figure 3k1), which undergoes dehydroxylation and ejects the secondary OH group (step k1 → l1 → m1), forming a C=C bond at the α–β position to yield CrH (Figure 3m1). The dissociated OH group can either scavenge a surface-adsorbed H atom (i.e., step m1 → n1 → o1) or abstract H from the α C of another AcH or 3HBtL. H abstraction by OH, instead of by O_latt_, can be viewed as a type of base-catalyzed mechanism. It reduces the activation barrier compared to those listed in Table 1 (steps b → c → d and i1 → j1 → k1) by 0.20 and 0.22 eV, respectively, which further facilitates the condensation reaction. Effective activation of C–H bonds by surface OH groups instead of directly by the catalyst has been previously suggested for aqueous phase reactions on metals.^64,65^

From co-adsorbed CrH and H_2_O at a combined 1/2 ML (Figure 3o1), CrH desorbs with a barrier of 0.93 eV (step o1 → p1, compared to the desorption of an isolated CrH, with −Δ*E*_ads_ = 0.71 eV), followed by the desorption of H_2_O. A plot of the differential adsorption energy of CrH with respect to coverage indicates interaction between CrH molecules in the absence of water to be attractive by 0.20 eV as the coverage increases up to 1/2 ML according to optB86b-vdW, but purely repulsive according to GGA-PW91 (Figure S2 in the SI).

Overall, our calculations show that the activation barriers for all the surface elementary steps are 0.74 eV or less (or 0.52 eV or less based on H abstraction by OH), which are all sufficiently small to be accessible at ambient temperature. State o1 has the lowest energy in the mechanism, at −1.70 eV below gas-phase AcH, indicating a significantly greater driving force for the aldol condensation of AcH on CeO_2_(111) than that in the gas phase. This, together with the moderate activation barriers, suggests that the surface reaction should proceed rapidly, resulting in a buildup of CrH and water, which is consistent with our DRIFTS observation.

The above mechanism produces *trans*-CrH. The *cis* isomer is calculated to be 0.12 eV less stable in the gas phase, which suggests that it may form in the reaction, too. The formation of the different isomers has been considered only in a few previous studies,^63,66^ but they did not clarify what controls the stereoisomerism. We propose that the bifurcation in the reaction mechanism occurs at the C–C coupling step. The specific steps after the bifurcation (starting at state e) are listed in the lower half of Table 1, with snapshots of the reaction intermediates shown in Figure S3 in the SI. Depending on how the second AcH approaches the Enl, either of the two 3HBtL enantiomers can form (cf. Figure 3i1, Figure S3i2), leading to *trans*-CrH (Figure 3z1) or *cis*-CrH (Figure S3z2), respectively. The energy profile for the *cis* mechanism is plotted in Figure 2, which is comparable to that of the *trans* mechanism. We conclude that the partition between *cis*- and *trans*-CrH is thermally equilibrated at ambient temperature, with the latter expected to be the dominant species. Previously, Rekoske and Barteau reported that *cis-* and *trans-*CrH were produced in essentially thermally equilibrated proportions in the aldol condensation of AcH on anatase TiO_2_ at elevated temperatures.^66^

### Proposed Mechanism on Partially Reduced CeO_2-x_(111)

Previous studies of the AcH reaction on CeNPs, whether reduced or not, have usually assumed V_O_ to be required by the reaction.^28,30^ Both theoretical calculations^67−69^ and experimental evidence^70^ have shown that V_O_ is located preferentially in the subsurface (in the bottom of the top tri-layer, i.e., the third atomic layer) on extended (111) facets of ceria. The exchange of V_O_ between the surface and subsurface locations is calculated to have modest activation barriers,^68,71^ and the migration of the V_O_ is tied to the movement of the associated polarons.^72^ The presence of an adsorbate that interacts strongly with V_O_ (such as carbonyl compounds) could enhance the exchange process,^35^ causing subsurface V_O_ to surface and be involved in surface reactions. For a CeO_2–*x*_(111) exposed to a finite *p*_AcH_, therefore, alternative reaction mechanisms based on AcH interacting with V_O_ could be operative. The subtle differences revealed by our in situ DRIFTS spectra between the oxidized and partially reduced o-CeNPs also suggest a separate pathway on reduced surface sites.

We previously investigated V_O_-based mechanisms for aldol condensation of AcH on CeO_2–*x*_(111) theoretically. Two variations were considered: a dimer V_O_ mechanism, which involves Enl/V_O_ coupling to an adjacent AcH/V_O_ (designated mechanism A in ref (35)); and a point V_O_ mechanism involving Enl/V_O_ coupling to an AcH on a nearby stoichiometric site (mechanism C in ref (35)). The former has a maximum barrier of 0.88 eV, whereas the latter has a maximum barrier of 1.20 eV. Both mechanisms are desorption-limited by CrH/V_O_, to a much higher temperature than CrH is on a stoichiometric site.

**Figure 4:**
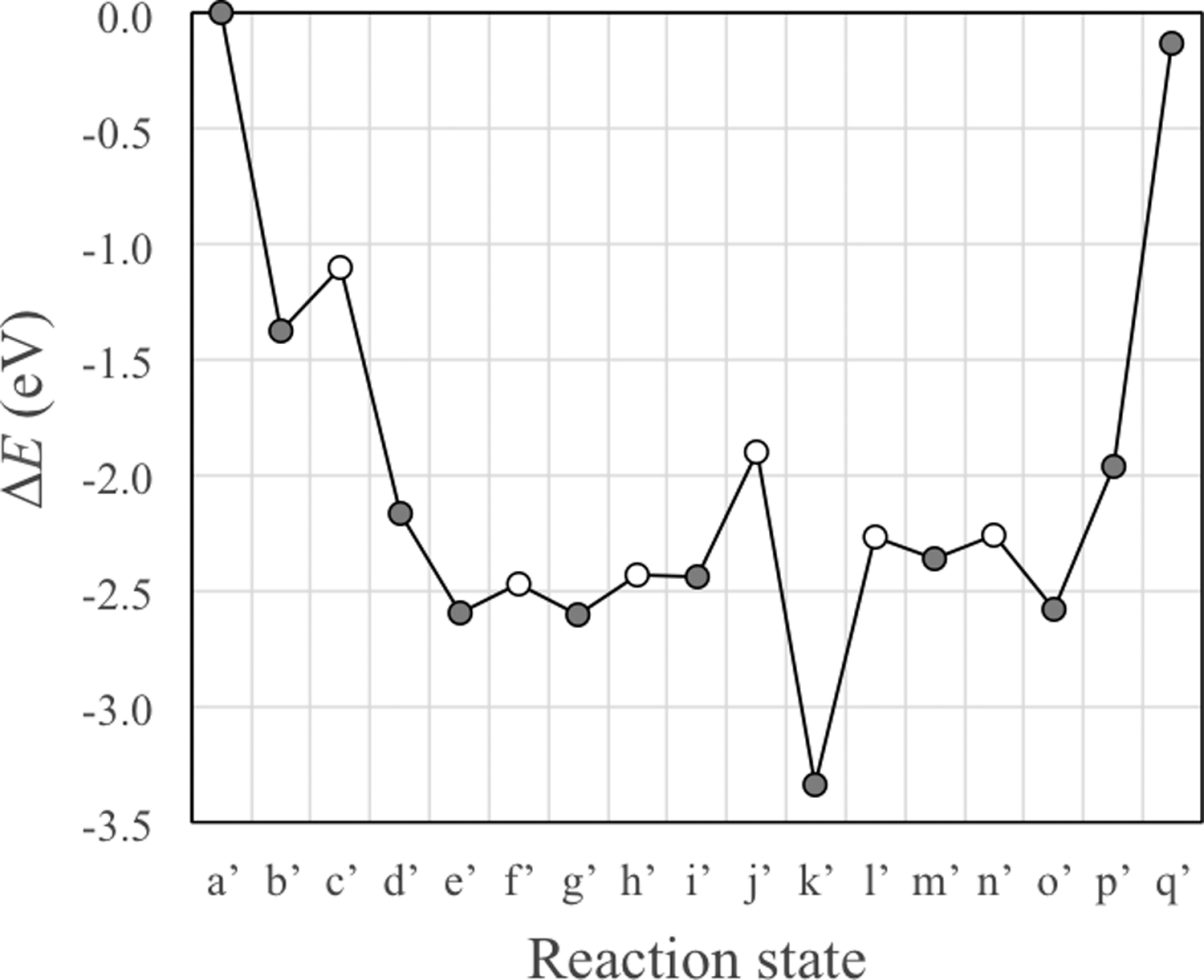
Reaction energy profiles for the aldol condensation of AcH to form *trans*-CrH on an oxygen vacancy on CeO_2_(111). The states along the reaction coordinate are (a′) AcH_(g)_, (b′) AcH/V_O_; (c′) TS of the first α C–H scission; (d′) Enl/V_O_ + H; (e′) the second AcH co-adsorbed with Enl/V_O_ + H; (f′) TS of the C–C coupling; (g′) CH_3_CHOCH_2_CHO/V_O_ + H; (h′) TS of hydrogenation; (i′) 3HBtL/V_O_; (j′) TS of the second α C–H scission; (k′) CH_3_CHOHCHCHO/V_O_ + H; (l′) TS of dehydroxylation; (m′) CrH/V_O_ + OH + H; (n′) TS of H_2_O formation; (o′) CrH/V_O_ + H_2_O; (p′) CrH/V_O_ + H_2_O_(g)_; and (q′) CrH_(g)_ + H_2_O_(g)_. Filled dots represent stable intermediates, and open dots represent TSs.

**Figure 5:**
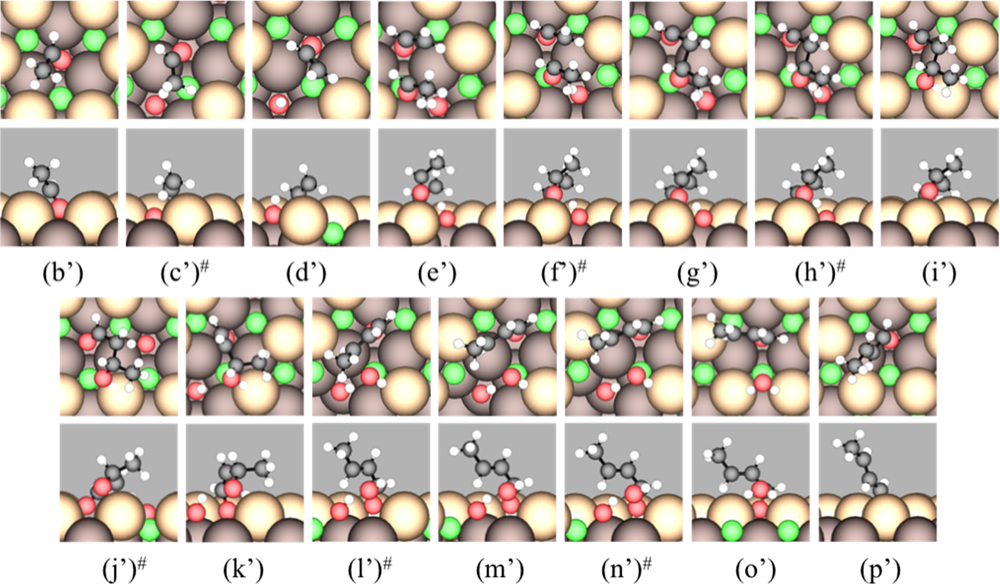
Top (upper panels) and side (lower panels) views of stable intermediates and TSs (labeled #) in the proposed mechanism for the aldol condensation of AcH to form CrH on an oxygen vacancy (V_O_) on CeO_2_(111). The labels correspond to those shown in Figure 4. The states shown are (b′) AcH/V_O_; (c′) TS of the first α C–H scission; (d′) Enl/V_O_ + H; (e′) AcH + Enl/V_O_ + H; (f′) TS of the C–C coupling; (g′) CH_3_CHOCH_2_CHO/V_O_ + H; (h′) TS of hydrogenation; (i′) 3HBtL/V_O_; (j′) TS of the second α C-H scission; (k′) CH_3_CHOHCHCHO/V_O_ + H; (l′) TS of dehydroxylation; (m′) CrH/V_O_ + OH + H; (n′) TS of H_2_O formation; (o′) CrH/V_O_ + H_2_O; and (p′) CrH/V_O_. Color code: green = lattice Ce, light brown = surface lattice O, dark brown = subsurface lattice O, red = O in molecules, black = C, and white = H. Surface lattice O atoms bonded to C or H atoms in the molecules are considered part of the molecules.

Given the low probability of Vo dimer formation at ambient temperature^69,73^ and given that a finite *p*_AcH_ exists in our experiment, we reconsider the point V_O_ mechanism (for brevity, for the formation of *trans*-CrH only). A lower barrier pathway is found than the one previously reported by us due to differences in how a second H is removed from the α C.^35^ The reaction energy profile is plotted in Figure 4, with snapshots of the reaction intermediates shown in Figure 5. The enolization (step b′ → c′ → d′, Table 2) and C–C coupling (step e′ → f′ → g′) steps are both facile, producing the adduct CH_3_CHOCH_2_CHO/V_O_ (Figure 5g′). As on stoichiometric sites, we capture the TSs for (i) direct intra-molecular H shift in CH_3_CHOCH_2_CHO/V_O_, (ii) α C–H bond scission in CH_3_CHOCH_2_CHO/V_O_, and (iii) α C–H bond scission following the formation of 3HBtL/V_O_ (Figure 5i′). The energies of the TSs are, relative to the last one, 0.30, 0.09, and 0.00 eV, i.e., in the same order as those on stoichiometric sites but with a smaller spread. Therefore, the Vo mechanism is constructed based on 3HBtL/V_O_ undergoing the second α C–H bond scission (step i′ → j′ → k′).

**Table 2:** DFT-Calculated Activation Barrier (*E*_a_, in eV) and Reaction Energy (Δ*E*_rxn_, in eV) for the Elementary Steps in the Proposed Mechanism for the Aldol Condensation of AcH on CeO_2_(111) with an Oxygen Vacancy (V_O_)^a^

label	elementary step	*E*_a_	Δ*E*_rxn_
a′ → b′	AcH_(g)_ → AcH/Vo	0^b^	–1.09^c^
b′ → c′ → d′	AcH/Vo → Enl/Vo + H	0.28	–0.79
e′ → f′ → g′	AcH + Enl/Vo → CH_3_CHOCH_2_CHO/Vo (+ H)	0.13	–0.01
g′ → h′ → i′	CH_3_CHOCH_2_CHO/Vo + H → 3HBtL/Vo	0.17	+0.16
i′ → j′ → k′	3HBtL/Vo → CH_3_CHOHCHCHO/Vo + H	0.54	–0.90
k′ → l′ → m′	CH_3_CHOHCHCHO/Vo → CrH/V_O_ + OH (+ H)	1.07	+0.98
m′ → n′ → o′	OH + H → H_2_O (+ CrH)	0.10^d^	–0.22
o′ → p′	H_2_O → H_2_O_(g)_ (+ CrH)	0.61^b^	+0.61^c^
p′ → q′	CrH/Vo → CrH_(g)_ + Vo	1.84^b^	+1.84^c^

a *E*_a_ and Δ*E*_rxn_ are based on GGA-PW91 electronic energies (*U*_eff_ = 2 eV) without ZPE correction. Step labels refer to Figure 4. Species shown in parentheses are co-adsorbed and are not directly involved in the given steps. Free sites are omitted from the description.

b Adsorption assumed to be barrier-less and desorption assumed to have no kinetic barrier in addition to the thermodynamic barrier.

c Corrected for vdW interactions based on optB86b-vdW results.

d Minimal calculated barrier, replaced with a value representing the OH diffusion barrier.

Pathways (i–iii) outlined above all end in the formation of CH_3_CHOHCHCHO/V_O_ (Figure 5k′), which preferentially adopts a position in which the interior OH group engages in hydrogen bonding with the nearest O_latt_ (dOH–O_latt_ = 1.64 Å) and, to a lesser extent, with a surface H atom (dH*–OH = 2.15 Å) such as the one dissociated in the previous step, i′ → j′ → k′. The dehydroxylation of this species faces a much higher activation barrier (*E_a_* = 1.07 eV) than on stoichiometric sites because it needs to shift the C=C bond from the carbonyl-α position to the α–β position and restore the terminal C–O as a carbonyl group. At ambient temperature this barrier is not surmountable, so CH_3_CHOHCHCHO/V_O_ becomes the energy sink in the V_O_ mechanism.

One difference between the reaction energy profiles on stoichiometric sites and on V_O_ is that the two enolization barriers (steps b′ → c′ and i′ → j′) are both notably lower on V_O_, driven by the fact that the resulting species, Enl/V_O_ (Figure 5d′) and CH_3_CHOHCHCHO/V_O_ (Figure 5k′) are much more stable relative to their precursors, AcH/V_O_ and 3HBtl/V_O_, than their counterparts on stoichiometric sites. We attribute it to the carbonyl O being stabilized more effectively by V_O_ than by occluded lattice Ce on a stoichiometric surface, which allows the carbonyl C=O bond to re-hybridize into a C–O single bond more effectively than on a stoichiometric surface.

### Simulated Infrared Spectra

**Figure 6:**
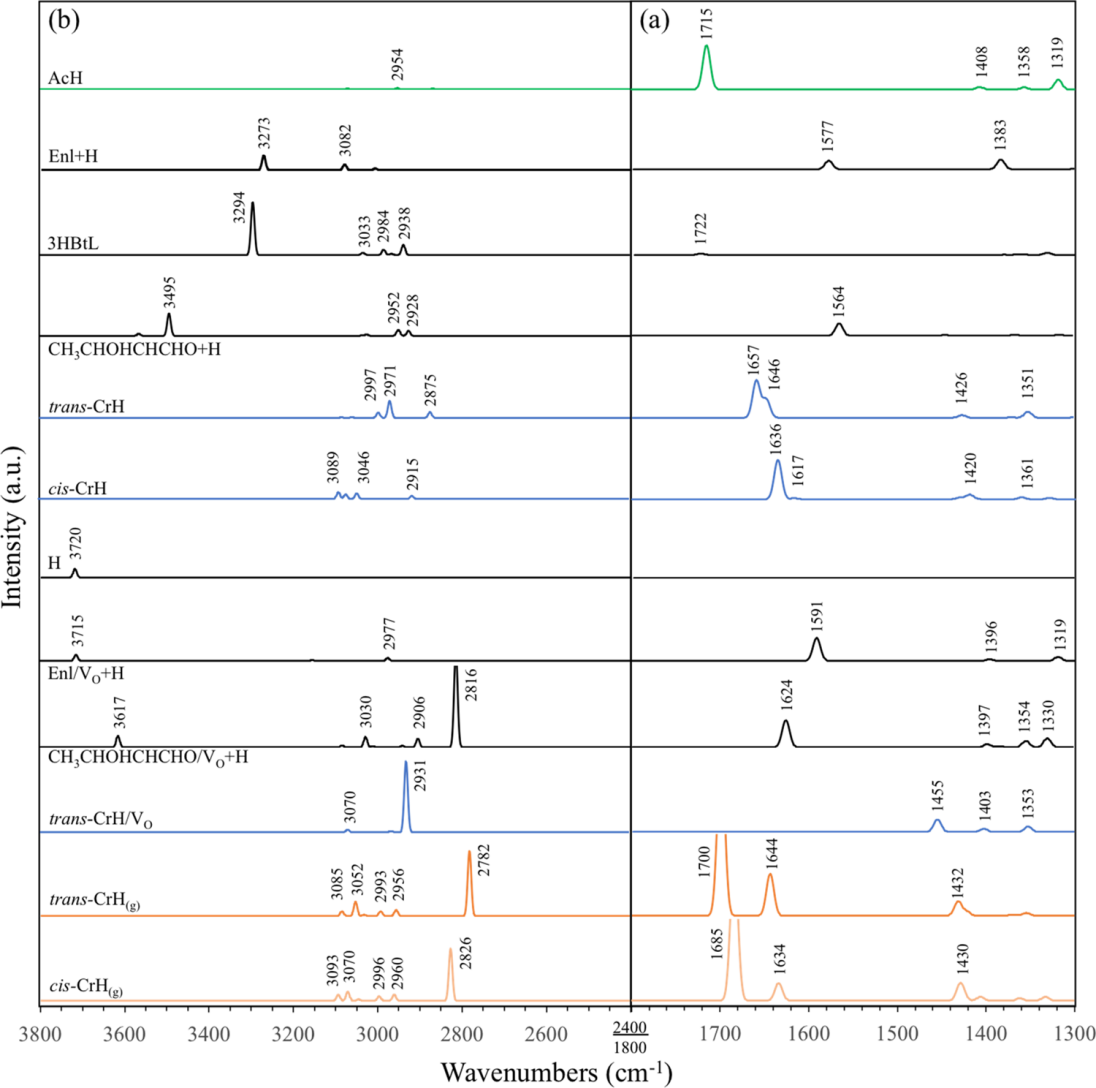
Simulated IR spectra of key surface species in the proposed mechanism for the aldol condensation of AcH to CrH on CeO_2_(111), calculated with *U*_eff_ = 2 eV: (a) 1300–1800 cm^–1^; (b) 2400–3800 cm^–1^. The spectra of gas-phase CrH are included at the bottom for comparison.

We furthermore simulate the IR spectra for key species in the proposed reaction mechanisms (Figure 6) to be compared with the DRIFTS spectra to further verify the validity of our proposed mechanisms. On stoichiometric CeO_2_(111), these species include the reactant, AcH (Figure 3b); the intermediates Enl + H (Figure 3d), 3HBtL (Figure 3i1), and CH_3_CHOHCHCHO + H (Figure 3k1); the products, *trans*-CrH (Figure 3z1), *cis*-CrH (Figure S3z2); and an isolated H. On CeO_2_(111) with an oxygen vacancy, these include Enl/V_O_ + H (Figure 5d′), CH_3_CHOHCHCHO/V + H (Figure 5k′), and *trans*-CrH/V_O_ (Figure 5p′). A corresponding set of spectra calculated using *U*_eff_ = 5 eV is included in Figure S4 in the SI to illustrate the small effects of this parameter on the vibrational frequencies of the surface intermediates in this reaction system.

Upon adsorption from the gas phase into the η^1^ state, the carbonyl C=O bonds of AcH, 3HBtL, and *trans*-CrH slightly lengthen, and the frequencies of the νC=O modes of these molecules are lowered by 30–50 cm^–1^ (cf. Table S2 in the SI), which indicates that molecular adsorption via the carbonyl O on a 3fc site, despite the fact that Ce atoms are occluded by O_latt_ atoms, involves significant electronic interaction. The softening of the νC=O mode is consistent with the observation of Rekoske and Barteau.^34^ AcH has a strong IR-active νC=O mode at 1715 cm^–1^, whereas *trans*-CrH has a main peak at 1657 cm^–1^ with a weaker adjacent peak at 1646 cm^–1^. The 1657 cm^–1^ mode is identified to be νC=O, and the 1646 cm^–1^ mode identified to be νC=C. The latter frequency is nearly identical to the frequency of the νC=C mode in the gas phase. These modes match closely the major peaks observed in DRIFTS (Figure 1, right panels), which supports the identification of *trans*-CrH as the main reaction product on the o-CeNPs and demonstrates CrH desorp is not facile at ambient temperature. Increasing the coverage of *trans*-CrH from 1/4 to 1/2 ML increases the intensities of these modes but does not affect the frequencies notably (not shown). Unlike the reactant or product, Enl + H, 3HBtL, and CH_3_CHOHCHCHO have no strong IR-active mode in the 1300–1800 cm^–1^ range (Figure 6a).

*cis*-CrH is distinguished from *trans*-CrH on CeO_2_(111) by the fact that its νC=O and νC=C stretches are calculated to be located at 1636 and 1617 cm^–1^, respectively, which are softer than the corresponding modes in *trans*-CrH. Given a calculated difference in energy of 0.04 eV between the two isomers in the η^1^ state, the coverage of *cis*-CrH is estimated to be less than 20% of that of *trans*-CrH, based on a Boltzmann distribution at 298 K. This means that the IR modes of *cis*-CrH, particularly the main νC=O mode that is very close to the νC=C mode of *trans*-CrH, is unlikely to be identifiable in the experiment.

Among the species in the V_O_ mechanism, AcH/V_O_ has no IR-active mode in 1500–1800 cm^–1^. Actual observation of AcH/V_O_ would be difficult because it has an even lower activation barrier of enolization than that of AcH on stoichiometric sites. The vacancy-stabilized Enl/V_O_ does have a strong mode in this range (1591 cm^–1^) as previously reported by us under UHV due to its νC=C mode.^32^ However, it does not correspond closely to any of the prominent peaks observed in DRIFTS, nor do we expect Enl/V_O_ to be observable due to its reactivity. *trans*-CrH/V_O_, unlike CrH but like AcH/V_O_, possesses no strong IR-active mode around 1600 cm^–1^. Overall, the νC=O mode of the terminal carbonyl bond, whether in the reactant, intermediates, or product, is red-shifted to well below 1700 cm^–1^ due to strong interaction with V_O_ (Table S2).

On the other hand, CH_3_CHOHCHCHO/V_O_ + H, which is the minimum-energy species in our proposed V_O_ mechanism (Figure 5k′), is predicted to have a prominent mode at 1624 cm^–1^ due to the stretch of the C–C bond between the carbonyl and α C atoms. The vibrational frequency and bond length (dC–C = 1.35 Å) suggest that it is a C=C double bond, for the same reason that the C–C bond in Enl/V_O_ is a double bond. This mode matches closely the 1620 cm^–1^ mode in DRIFTS (which originates at 1610 cm^–1^ at low coverage). Based on these considerations, we identify this mode with CH_3_CHOHCHCHO/V_O_. The stronger intensity of this mode observed on the partially reduced o-CeNPs is thus consistent with a species adsorbed on a reduced site. Its presence on the oxidized sample (Figure 1, right panel) suggests that the latter is not entirely free of oxygen vacancies. The facile formation and the IR-active νC=C mode of CH_3_CHOHCHCHO/V_O_ suggest that IR coupled with AcH adsorption may be a viable probe for detecting the presence of oxygen vacancies in the surface and near-surface regions of ceria nanoparticles. This provides an alternative method to using Raman spectroscopy to detect, e.g., V_O_-bound O_2_ species, the outcome of which appears to be sensitive to synthesis and treatment of ceria samples, particularly regarding the prevalence of surface V_O_ on (111) facets.^37,70,74^

All of the organic species included in Figure 6 except Enl have terminal methyl groups, which have asymmetric and symmetric bending modes located somewhere around 1350–1460 cm^–1^. These modes are much weaker than the νC=O or νC=C modes mentioned above. The calculated δ_as_CH_3_ and δ_s_CH_3_ modes of *trans*-CrH, 1426 and 1351 cm^–1^, match the observed 1430 and 1354 cm^–1^ features most closely. Other species that have been concluded to be present on the surface, including AcH and CH_3_CHOHCHCHO/V_O_, also contribute some visible signals in this range. AcH, Enl/V_O_, and CH_3_CHOHCHCHO/V_O_ also possess minor modes in 1300–1350 cm^–1^ involving the rocking of their interior CH groups.

The terminal methyl groups also exhibit asymmetric and symmetric stretching modes located around 2900–3100 cm^–1^ (Figure 6b). According to our calculations, the species that contribute IR activity in this range are *trans*-CrH (ν_as_CH_3_ at 2997 cm^–1^ and ν_s_CH_3_ at 2971 cm^–1^) and CH_3_CHOHCHCHO/V_O_ (ν_as_CH_3_ at 3030 cm^–1^). The strong mode at 2931 cm^–1^ for *trans*-CrH/V_O_ is also ν_s_CH_3_. The CH_3_ modes of AcH have vanishing intensities, which is at discrepancy with the experiment.^32^ CH_3_CHOHCHCHO/V_O_ also possesses some CH stretching modes for the interior C atoms at the lower end of this range, the most intense of which being νC_β_H at 2906 cm^–1^.

The IR-active modes above 3100 cm^–1^ are mainly attributed by OH-related moieties. An isolated adsorbed H atom on CeO_2_(111) is calculated to have an O_latt_–H stretch at 3720 cm^–1^, which we take to correspond to the negative 3678 cm^–1^ feature in DRIFTS (Figure 1, left panels). Its gradual disappearance implies that at least some of the product water molecules aggregate over the hydroxylated surface areas. Simulating the IR response of a disordered domain of adsorbed water is outside the scope of this study. The atomic H that is co-adsorbed with Enl/V_O_ has an O_latt_–H stretch of 3715 cm^–1^.^32^ On the other hand, the atomic H co-adsorbed with CH_3_CHOHCHCHO/V_O_ has a red-shifted O_latt_–H stretch at 3617 cm^–1^ due to close proximity to the interior hydroxyl group of the molecule (with dO_latt_–H = 0.98 Å, compared to 0.97 Å for the isolated H or H co-adsorbed with Enl/V_O_). We take it to correspond to the 3592 cm^–1^ band in DRIFTS. The fact that it is separate from the broad feature at 3000–3500 cm^–1^ suggests that the organic species, possibly mixed with some water molecules, exist in a separate domain. Incidentally, the OH stretch of the interior hydroxyl group of CH_3_CHOHCHCHO/V_O_ is calculated to be strongly IR-active at a lower frequency of 2816 cm^–1^. A corresponding feature may be the 2775 cm^–1^ band in DRIFTS.

## Discussion

Our findings conclusively show that under a finite partial pressure, the aldol condensation of AcH occurs readily on the (111) facet of ceria at ambient temperature with apparently complete selectivity to CrH. This is consistent with previous infrared and mass spectroscopy studies of AcH adsorption on different oxides including CeO_2_ and TiO_2_, which reported CrH formation with high selectivity near or below ambient temperature.^14,17,28,34,59^ Our DFT calculations indicate a sizable difference between the desorption barrier for CrH and the activation barriers of the two enolization steps (in AcH and 3HBtL, steps b → c → d and i1 → j1 → k1). To further ascertain the role of CrH desorption in this reaction, we compare the free energy activation barriers (*G_a_* = *G*_TS_ – *G*_IS_) of several key steps in Table 3. The free energy contributions (δ*G*) of the ISs and TSs of these steps are estimated by taking the vibrational contributions into account, in line with high surface coverages. The exception is the TS of CrH desorption, which is taken to be an isolated CrH molecule behaving as an ideal 2D gas at a pressure of 0.1 bar.^75^

**Table 3:** Comparison of Various Measures of the Activation Barriers (*E_a_*: Electronic Energy Barrier; *E_a_*^ZPE^: *E_a_* Corrected by Zero Point Energies; *G_a_*: Free Energy Barrier; All in eV) for the Key Steps in the Aldol Condensation of AcH on CeO_2_(111)

	AcH enolization (step b → c → d)		3HBtL enolization (step i1 → j1 → k1)		CrH desorption (step o1 → p1)	
*E_a_*	0.52		0.74		0.93	
*E_a_*^ZPE^	0.41		0.61		0.88	
	298 K	523 K	298 K	523 K	298 K	523 K
*G_a_*	0.49	0.59	0.66	0.72	0.80	0.74

**Figure 7:**
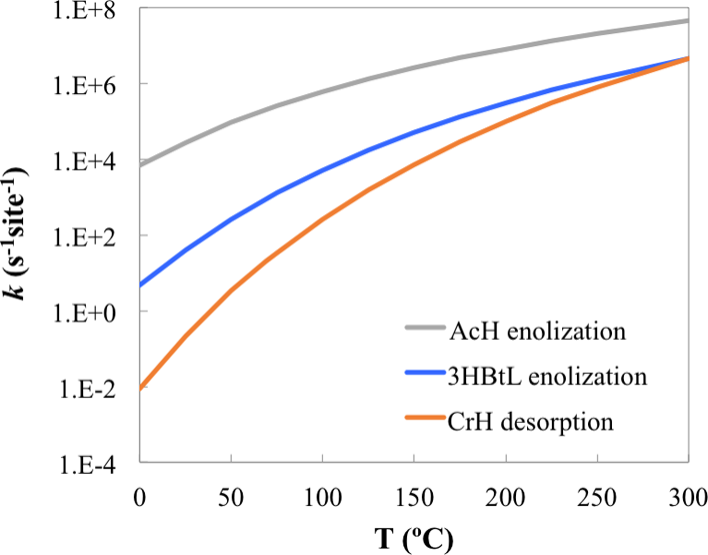
Estimated forward rate constants (*k*) of the three steps listed in Table 3.

The *G_a_* values of both surface enolization steps are small, consistent with observed ease of the surface reaction. The *G_a_* of CrH desorption exceeds those of the two surface steps, from ambient temperature all the way to near the higher end of reaction temperatures tested in the literature (ca. 300 °C). Previously, Mann et al. found CrH desorption to occur starting at ca. 75 °C in the temperature-programmed reaction of AcH over o-CeNPs,^28^ which implies the stability of CrH to be ca. −0.9 eV, with which the estimated *G_a_* in Table 3 is consistent. The associated forward rate constants, calculated as , are plotted as a function of temperature in Figure 7, which shows the *k* of CrH desorption to be the lowest of the three steps in the whole range of temperature considered. The differences could be further amplified by possible H abstraction pathways catalyzed by OH groups, which lowers the barriers of the enolization steps compared to abstraction by O_latt_. Therefore, we conclude that the reaction mechanism as proposed herein is desorption-limited by CrH to a significant temperature. Assuming θ_CrH_ ≈ 1, the predicted rate of CrH desorption exceeds 1.0 s^–1^ site^–1^ at ca. 50 °C (Figure 7), somewhat lower than where CrH desorption becaome significant in the study of Mann et al.,^28^ when coverage effect for CrH is not considered. Clearly, without accounting for vdW contributions to adsorption, the reaction would not be predicted as desorption-limited by CrH (cf. Figure S1).

To allow the reaction to turn over on the o-CeNPs, it would be necessary to facilitate the desorption of CrH. Raising the reaction temperature is one way, although doing so would increase the likelihood of secondary reactions.^66^ For instance, Mann et al. reported that the amount of carbon captured by o-CeNPs became significant starting somewhere between 470 and 570 K.^28^ Another way to enhance CrH desorption is to carry out the reaction in a suitable solvent. We estimate that a prospective solvent can lift the desorption limitation and shift the kinetic bottleneck to a surface reaction step (i.e., enolization of 3HBtL) by solvating CrH by ca. 0.4 eV (or 9 kcal/mol) or more. A further strategy to encourage product desorption is to chemically reduce the reactive C=O and C=C bonds.^26,62^

Unlike many catalytic reactions, the product of the aldol condensation of AcH is itself an aldehyde and can undergo further aldol condensation. A buildup of CrH coverage not only takes up sites needed to activate AcH (cf. Figure 1, AcH begins to accumulate following the buildup of CrH coverage) but also increases the possibility of poly-condensation. With poly-condensation, ensuing products are less and less volatile and therefore deactivate the catalyst. Those studies in the literature that used differential conversion in flow reactors were able to obtain steady-state turnover, although strong deactivation and carbon uptake were evident in an initial period of time on stream.^17,66^ If a higher conversion or higher pressure was used, continuing deactivation occurred without a steady state being reached.^26^

One can derive an analytical rate law based on a lumped version of the mechanism presented in Table 1 (see Table 4), with all steps except one taken to be quasi-equilibrated, to estimate the dependence of the reaction kinetics on *p*_AcH_, i.e., what *n* is equal to in *r* ∝ *p*_AcH_^*n*^.

**Table 4:** Abbreviated Aldol Condensation Mechanism of AcH to CrH on CeO_2_(111)^a^

1.	AcH_(g)_ + * ↔ AcH*
2.	AcH* + * ↔ Enl**
3.	AcH* + Enl** ↔ 3HBtL** + *
4.	3HBtL** ↔ CrH* + H_2_O*
5.	CrH* ↔ CrH_(g)_ + *
6.	H_2_O* ↔ H_2_O_(g)_ + *

a * represents a surface site.

If the desorption of CrH (step 5) is rate-determining, then the reaction rate is . When the surface is largely clean (θ_*_ → 1), . The kinetics then depends on whether the reaction is overall equilibrated: If the reaction is overall equilibrated, *p*_H_2_O_ ∝ *p*_AcH_ and *n* → 1, while if the reaction is far from being equilibrated and if we take *p*_H_2_O_ to be independent of *p*_AcH_ and *p*_H_2_O_ ≪ *p_AcH_*, then *n* → 2. When the surface is mostly occupied (θ_*_ → 0),
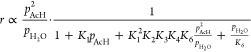
It can be shown that *n* → 0 regardless of whether the overall reaction is equilibrated.

If, on the other hand, the desorption limitation is lifted, the next most likely rate-determining step is step 4, which is the activation of 3HBtL. In this case, the reaction exhibits a kinetic isotope effect (KIE), and the overall rate is *r* = *k*_4_*K*_1_^2^*K*_2_*K*_3_*p*_AcH_^2^θ_*_^2^. It can be shown that the limiting values of *n* are 0 and 2, corresponding to θ_*_ → 0 and θ_*_ → 1, respectively. This implies that KIE, when observed, could occur with a reaction order ranging from 0 to 2, depending on how the reaction is carried out.

To summarize,

- *n* → 0 indicates a highly covered catalyst surface (θ_*_ → 0), regardless of which step is rate-determining, or whether the overall reaction is equilibrated;
- A clear non-zero order occurs on a largely clean surface (θ_*_ → 1), with *n* → 1 if the reaction is desorption-limited and equilibrated overall and *n* → 2 if the reaction is desorption-limited but not equilibrated or if the reaction is not desorption-limited.

Recently, Li et al. reported that the rate of aldol condensation of cyclopentanone exhibited second-order kinetics at low cyclopentanone concentrations when carried out in a batch reactor with cyclohexane as solvent at 473 K over certain ceria nanoshapes, including o-CeNPs. We interpret it as due to both an alleviation of desorption limitation and low activity of the reactant. As the reactant concentration increased, the reaction rate rapidly approached zero order on o-CeNPs.^76^ Young et al. reported the initial rates of aldol condensation of AcH over TiO_2_ at 553 K to be first order below 10 kPa of *p*_AcH_ but they deviated gradually toward zero order with increasing *p*_AcH_,^17^ which we suggest to be a consequence of an increasingly covered surface. The authors tested but did not detect any KIE, suggesting that the reaction was desorption-limited. On hydroxyapatite and MgO where adsorptions were stronger, the initial rates were nearly zero order across the whole range of *p*_AcH_ that they tested, reflecting rapid deactivation.^17^

Under desorption limitation, the proposed mechanism, which does not account for any poly-condensation product being an abundant surface species, does not reflect possible additional surface reactivity along such pathways. Poly-condensates may include long linear poly-enes that result from the repeated addition of enolized AcH to existing aldehydes, branched species that result from enolized 3HBtL undergoing C–C coupling, or ether or ester species due to carbonyl O, instead of enolized α C, acting as a nucleophile to attack other carbonyl groups. Conceivably, large poly-condensates can also be hydrolyzed via retro-aldol reactions to release smaller species when water is produced in forward condensation steps. The smaller of such poly-condensates, including 2,4-hexadienal, methyl vinyl ketone, and ethyl crotonate, were detected by Rekoske et al. over TiO_2_ at 523 K, totaling to with less than 5% of the selectivity.^66^ No such species is detected in our DRIFTS experiment, which may be due to a lack of sites for continued enolization of AcH. By not including secondary condensation pathways, the proposed mechanism does not account for the influence of poly-condensates on reaction kinetics. One may expect, nonetheless, the kinetics to have a zero dependence on *p*_AcH_ when poly-condensates become abundant on the ceria surface causing θ_*_ → 0.^66^

A variation on the CrH formation mechanism is what Wang et al. proposed for the aldol condensation of AcH on TiO_2_, in which 3HBtL was proposed to desorb from the oxide and equilibrate with CrH and H_2_O in the gas phase.^26^ This allowed the authors to rationalize observed first-order kinetics with KIE in their experiment. On CeO_2_(111), however, the calculated adsorption energy of 3HBtL (Table S1) is significantly more exothermic than that of AcH and CrH, suggesting a 3HBtL desorption pathway to be unlikely.

The current study, together with a body of experimental and computational work by our colleagues and ourselves over the past decade, has accumulated extensive insights into another important aspect of ceria catalysis, i.e., the role of oxygen vacancies, in this reaction system on CeO_2_(111).^28,31,32,35,52,77^ We now know that V_O_ is indispensable to CrH formation in UHV. V_O_ facilitates enolization of AcH (forming Enl/V_O_), but in UHV, because the enolate cannot undergo C–C coupling due to a lack of additional AcH to react with, it instead decomposes to C_2_H*_x_* species.^31,52^ A partial temperature ramp can generate a small amount of open, mobile V_O_ to dimerize with a V_O_ containing an existing Enl and form the necessary active site to enable C–C coupling when a second dosing of AcH is supplied.^35^ With a finite *p*_AcH_, a point V_O_, which is far more prevalent than a V_O_ dimer, is sufficient to allow AcH to couple with another AcH, but the reaction flux is trapped in the CH_3_CHOHCHCHO/V_O_ state. Eventually, over 600 K is needed to desorb CrH from CrH/V_O_.^35^ The extent to which V_O_ functions as a catalytically active site in this reaction on ceria is further limited by the fact that water, as a product of the reaction, can readily dissociate into pairs of adsorbed H atoms and annihilate surface V_O_.^71^ At sufficiently elevated temperature where atomic H can desorb as water, one should expect ceria to become partially reduced and multiple types of active site, including V_O_, to come into play and convolute the kinetic behavior of the reaction.

## Conclusions

By monitoring acetaldehyde (AcH) flown over ceria nano-octahedra (o-CeNPs) with in situ DRIFTS and by comparison with DFT-derived simulated IR spectra of relevant reaction intermediates on CeO_2_(111), we conclusively show the formation of crotonaldehyde (CrH) as the primary aldol condensation product of AcH at ambient temperature. A set of strong peaks at 1660 (νC=O) and 1640 (νC=C) cm^–1^ in DRIFTS is identified as *trans*-CrH adsorbed on stoichiometric CeO_2_(111). The buildup of CrH and water modes indicates the reaction to be desorption-limited at ambient temperature. A reaction mechanism with DFT-calculated parameters is proposed. Neither the AcH enolization step nor the C–C coupling step is calculated to have the highest activation barrier among the surface reaction steps in the mechanism, which belongs instead to the enolization of the aldol intermediate, 3-hydroxybutanal. While *cis*-CrH is also produced, facile kinetics allows the equilibration between the two isomers at ambient temperature, which favors the more stable *trans* isomer.

Besides the modes identified with AcH, CrH, and water, a peak at 1620 cm^–1^ is also clearly visible in DRIFTS. It is more prominent on the partially reduced o-CeNPs than on the oxidized o-CeNPs and develops even before CrH formation and AcH accumulation. A similar feature is seen in some previous studies of AcH adsorption on other oxides, but its assignment has never been clearly made before. Our DFT calculations show that an analogous AcH condensation mechanism is operative on an oxygen vacancy, although the reaction flux would be trapped in an intermediate state CH_3_CHOHCHCHO/V_O_, which is calculated to possess a strong IR-active νC=C mode at 1624 cm^–1^.

Overall, based on the evidence on CeO_2_ and other oxides, the aldol condensation of AcH may be a universal reaction on all oxides that exhibit both acid (i.e., metal cation) and base (i.e., oxygen anion) sites in suitable proximity on the surface. It proceeds rapidly at ambient temperature on CeO_2_(111), but the product CrH is desorption-limited in gas phase catalysis. Our proposed mechanism exhibits kinetic behavior that is consistent with measurements of the aldol condensation of AcH on ceria and other oxides. The results provide valuable insights to further catalyst development for this archetypical reaction system.

## Supporting Information Available

Tabulated adsorption energies of closed-shell species in the proposed mechanisms and characteristics of gas-phase vs adsorbed AcH, 3HBtL, and *trans*-CrH; plots of reaction energy profiles for the proposed *trans*-CrH mechanism calculated using different methods; differential adsorption energy of *trans*-CrH; snapshots of stable intermediates and transition states in the proposed mechanism for *cis*-CrH formation on CeO_2_(111); and simulated IR spectra calculated at *U*_eff_ = 5 eV (PDF)
