# Supplementary material for: Elucidating the Mechanism of Ambient-Temperature Aldol Condensation of Acetaldehyde on Ceria
Source: ACS Catal. 2021 Jun 30;11(14):8621–34. doi: 10.1021/acscatal.1c01216 (PMC8294007; doi:10.1021/acscatal.1c01216)
Supplement: Supplementary file 1 — cs1c01216_si_001.pdf [file cs1c01216_si_001.pdf]

Elucidating the Mechanism of Ambient-Temperature Aldol Condensation of Acetaldehyde on Ceria

Suman Bhasker-Ranganath,[†] *Md. Saeedur Rahman,*[†] *Chuanlin Zhao,*[†§] *Florencia Calaza,*[‡] *Zili Wu,*[||] *and Ye Xu*[†*]

[†] Cain Department of Chemical Engineering, Louisiana State University, Baton Rouge, Louisiana 70803, United States

[‡] Instituto de Desarrollo Tecnoloǵico para la Industria Química (INTEC), CONICET-UNL, Santa Fe 3000, Argentina

[||] Chemical Sciences Division, Oak Ridge National Laboratory, Oak Ridge, Tennessee 37831, United States

[§] Present affiliation: Department of Chemical Physics, School of Chemistry and Materials Science, University of Science and Technology of China, Hefei, Anhui 230026, China

Supporting Information

Table S1. DFT-calculated adsorption energies (ΔE_{ads}, in eV) for closed-shell species involved in the proposed mechanism for aldol condensation of AcH on CeO_2(111), at 1/4 ML coverage.

	GGA-PW91	GGA-PW91 (vdW corr'd)	optB86b-vdW
AcH	-0.30	-0.59	-0.59
3HBtL	-0.58	-	-1.28
trans-CrH	-0.24	-0.71	-0.71
cis-CrH	-0.32	-0.79	-0.74
H_2O	-0.52	-0.63	-0.69

Table S2. Lengths (d, in Å) of C=O and C-C bonds, and vibrational frequencies (ν, in cm^{-1}) of C=O bond in AcH, 3HBtL, and CrH in gas phase, and adsorbed in η^1 and V_O states on $CeO_2(111)$.

	Gas phase			η^1 state						V_O state					
				(U_{eff} = 2 eV)			(U_{eff} = 5 eV)			(U_{eff} = 2 eV)			(U_{eff} = 5 eV)		
	dC=O	dC-C	νC=O	dC=O	dC-C	νC=O	dC=O	dC-C	νC=O	dC=O	dC-C	νC=O	dC=O	dC-C	νC=O
AcH	1.22	1.50	1754	1.23	1.49	1715	1.23	1.49	1712	1.31	1.49	1249	1.25	1.46	1583
3HBtL	1.22	1.51	1750	1.22	1.51	1721	1.22	1.51	1721	1.30	1.48	1285	1.26	1.44	1596
		1.53			1.53			1.52			1.57			1.66	
		1.52			1.52			1.52			1.53			1.54	
trans-	1.23	1.46	1700	1.24	1.46	1656	1.24	1601	1655	1.32	1.41	1274	1.27	1.43	1487
CrH		1.35			1.35						1.37			1.36	
		1.49			1.49						1.49			1.48	

GGA-PW91 results. dC-C of 3HBtL and CrH are listed in the order of C-C_α, C_α-C_β, C_β-C_γ, with the first C being that in the terminal C=O group (see Scheme 1 for labeling of the C atoms).

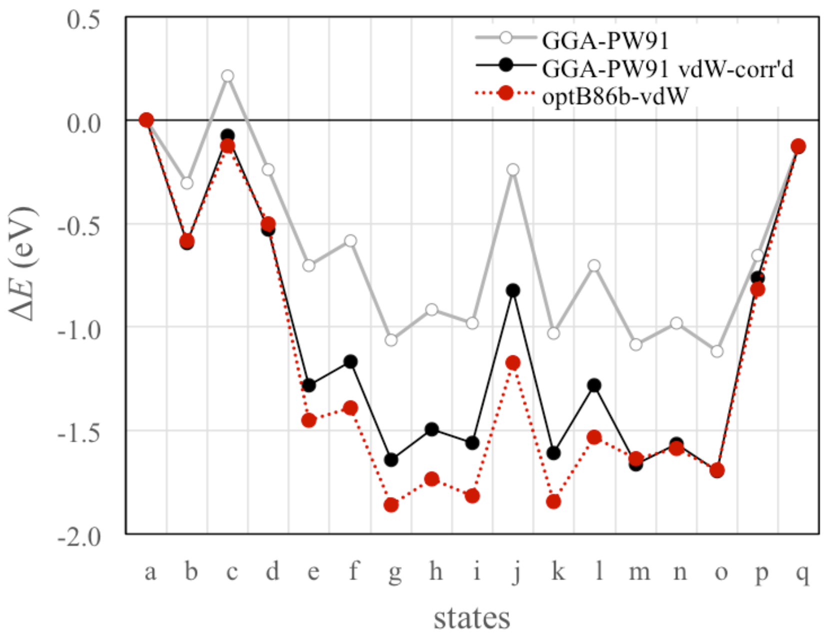

Figure S1. Reaction energy profiles for the proposed mechanism for aldol condensation of AcH to form *trans*-CrH on stoichiometric $CeO_2(111)$, calculated using GGA-PW91, GGA-PW91 with adsorptions of AcH, CrH, and H_2O corrected for vdW interactions, and optB86b-vdW, plotted together for comparison.

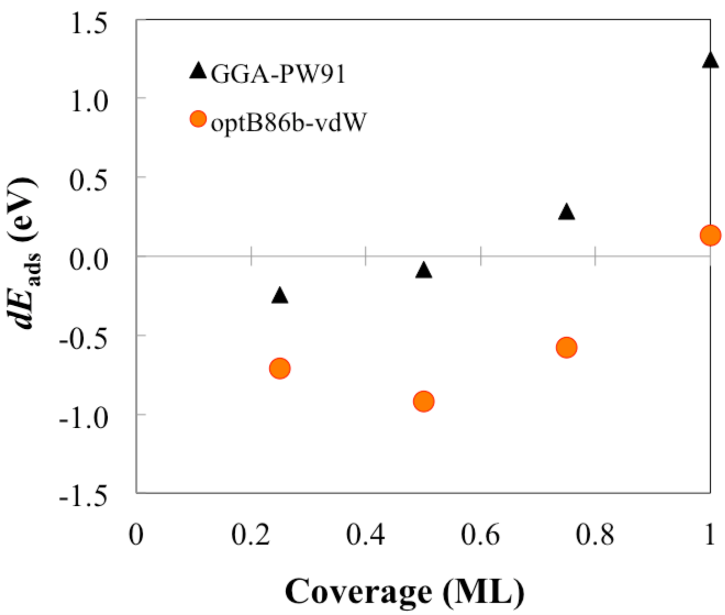

Figure S2. Differential adsorption energy (defined to be $dE_{ads} = E_{total\ (n+1)\ CrH} - E_{total\ (n)\ CrH} - E_{CrH(g)}$) of *trans*-CrH molecularly adsorbed on CeO_2(111), plotted against coverage. GGA-PW91 results are not corrected for vdW contributions.

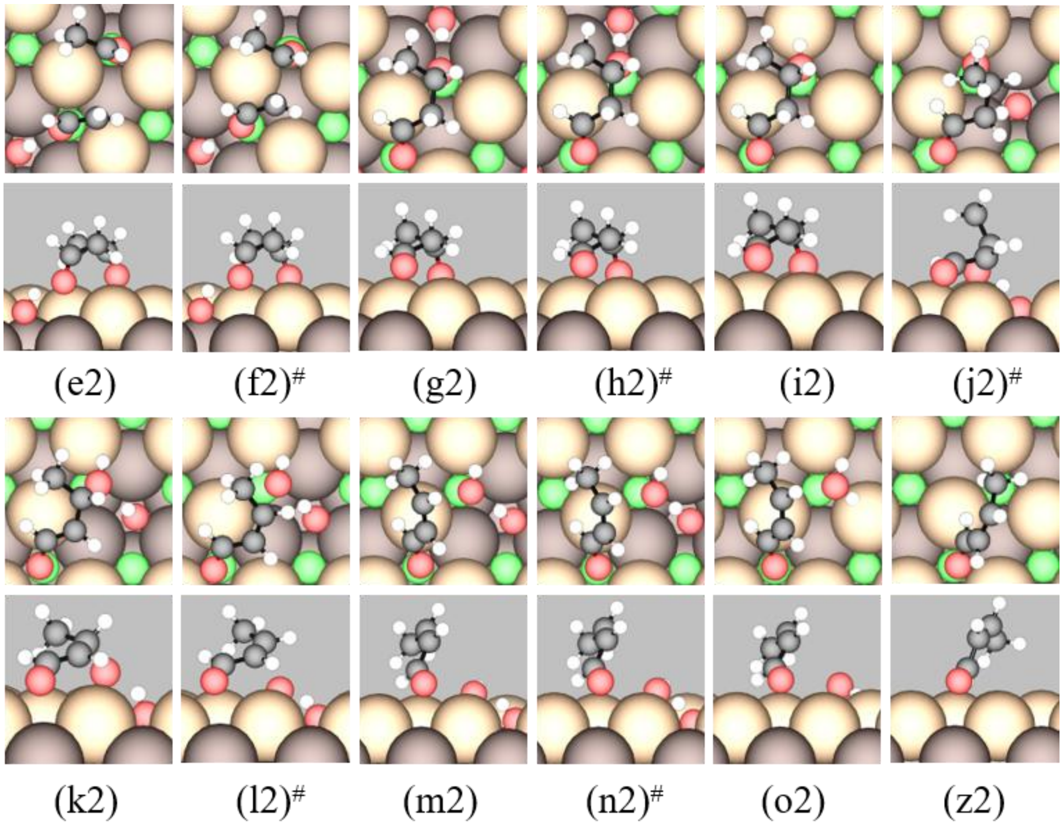

Figure S3. Top (upper panels) and side (lower panels) views of stable intermediates and TSs (labeled #) in the proposed mechanism for aldol condensation of AcH to *cis*-CrH on stoichiometric $CeO_2(111)$. The labels correspond to those in Table 1, starting where the bifurcation occurs. The states shown are: (e2) AcH + Enl + H; (f2) TS of C-C coupling of AcH and Enl; (g2) CH_3CHOCH_2CHO + H; (h2) TS of hydrogenation; (i2) (S)-3HBtL; (j2) TS of the second α C-H scission; (k2) $CH_3CHOHCHCHO$ + H; (2l) TS of dehydroxylation; (m2) CrH + OH + H; (n2) TS of H_2O formation; (o2) CrH + H_2O; and (z2) *cis*-CrH. Color code: Green=lattice Ce, light brown=surface lattice O, dark brown=subsurface lattice O, red=O in molecules, black=C, and white=H. Surface lattice O atoms bonded to C or H atoms in the molecules are considered part of the molecules.

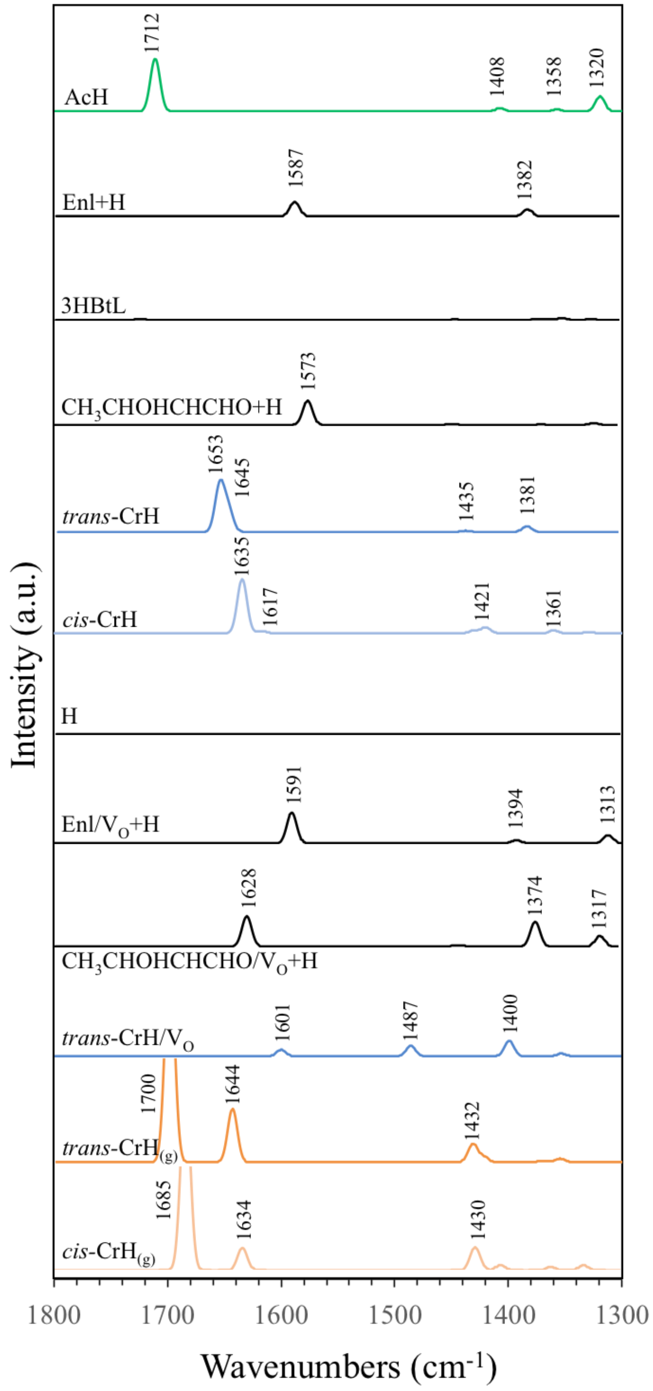

Figure S4. Simulated IR spectra of key surface species in the proposed mechanism for the aldol condensation of AcH to CrH on $CeO_2(111)$, calculated with U_{eff} = 5 eV. The lattice constant of bulk CeO_2 was calculated to be 5.494 Å at U_{eff} = 5 eV according to GGA-PW91.
